# Recent insights into the evolution of innate viral sensing in animals

**DOI:** 10.1016/j.mib.2014.05.010

**Published:** 2014-08

**Authors:** Samuel H Lewis, Darren J Obbard

**Affiliations:** Institute of Evolutionary Biology, and Centre for Immunity, Infection and Evolution, University of Edinburgh, Kings Buildings, EH9 3JT, United Kingdom

## Abstract

The evolution of viral sensors is likely to be shaped by the constraint imposed through high conservation of viral Pathogen-Associated Molecular Patterns (PAMPs), and by the potential for ‘arms race’ coevolution with more rapidly evolving viral proteins. Here we review the recent progress made in understanding the evolutionary history of two types of viral sensor, RNA helicases and Toll-like receptors. We find differences both in their rates of evolution, and in the levels of positive selection they experience. We suggest that positive selection has been the primary driver of the rapid evolution of the RNA helicases, while selective constraint has been a stronger influence shaping the slow evolution of the Toll-like receptors.

## Highlights

• Viral sensor evolution may be constrained by highly conserved viral immune-elicitors.
• Alternatively, viral sensor evolution may be driven by ‘arms race’ coevolution.
• We find viral-sensing Toll-like receptors evolve more slowly than other TLRs.
• In contrast, viral-sensing helicase related genes often evolve rapidly and adaptively.

**Current Opinion in Microbiology** 2014, **20**:170–175

This review comes from a themed issue on **Host**–**microbe interactions: viruses**

Edited by **Maria-Carla Saleh**

For a complete overview see the Issue and the Editorial

Available online 18th July 2014

**http://dx.doi.org/10.1016/j.mib.2014.05.010**

1369-5274/© 2014 The Authors. Published by Elsevier Ltd. This is an open access article under the CC BY license (http://creativecommons.org/licenses/by/3.0/).

## Introduction

Pathogens reduce host fitness, and thereby exert a strong and ubiquitous selective pressure on hosts that has led to the evolution of a range of immune responses. Immune responses are elicited when sensors detect the presence of pathogens through Pathogen-Associated Molecular Patterns (PAMPs) or through markers of pathogen-associated damage. However, viruses may be uniquely difficult to sense because they use the host's own machinery to replicate, and therefore present fewer exogenous elicitors to immune surveillance mechanisms. Innate antiviral responses are therefore often triggered by conserved signatures of viral nucleic acids, such as dsRNA or CpG dinucleotides, which lead to the activation of multiple downstream immune responses, such as the RNA interference pathway or the vertebrate interferon response.

The conserved nature of these viral PAMPs leads to contrasting predictions regarding the evolution of antiviral genes. On the one hand, sensing these ancient and conserved molecular signatures might be expected to constrain the evolution of viral sensors. On the other hand, viral suppression of the antiviral immune system may lead to rapid evolution of viral sensors, as is seen in some antiviral genes of *Drosophila* [1]. Such rapid evolution may be driven by a host-virus arms race, as viruses escape the host immune response by cleaving or blocking antiviral genes [2]. Mechanisms of viral sensing have recently been reviewed elsewhere [3]; here we summarise the recent progress that has been made in understanding how two important viral sensing mechanisms have evolved, focussing on both phylogenetic history and the ongoing natural selection that shapes antiviral responses of extant populations. We finish by weighing the relative contributions of positive selection and evolutionary constraint during the evolution of viral sensing.

## The phylogenetic distribution of viral sensing mechanisms

**Figure 1:**
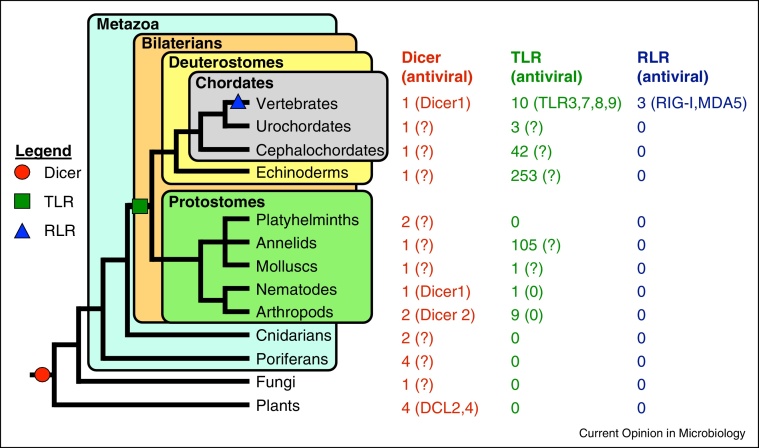
Phylogenetic distribution of viral sensing mechanisms. Gene family sizes are given, with validated antiviral genes in parentheses (0 = no antiviral genes, ? = antiviral function unknown). The three viral sensing mechanisms vary widely in their evolutionary ages: Dicer arose in the early Eukaryotes, whereas TLRs evolved in the early Bilateria, and RLRs first appeared in the vertebrates.

Although multiple protein families are known to act as viral sensors, many recent evolutionary studies have focussed on the Toll-like receptors (TLRs) and on receptors related to the RNA helicases, such as the Dicers and the RIG-I-like receptors (RLRs). Dicers act as sensors in the RNA interference (RNAi) pathway, binding dsRNA derived from the viral genome, replication intermediates or subgenomic products, and cleaving it into small RNAs that are ultimately used to target the virus or its transcripts for degradation. This is an ancient mechanism that probably arose prior to the most recent eukaryotic common ancestor over 1.5 billion years ago, and has since been conserved in all major eukaryotic lineages, including plants, fungi, ecdysozoa and vertebrates (illustrated in Figure 1) [4]. The helicase domain of the RLRs probably shares a common ancestor with that of Dicer [5], but on sensing viral dsRNA or other PAMPs, RLRs instead activate transcription factors such as nuclear factor-kappa B (NF-κB), and thereby induce the interferon pathway [6]. The RLRs also have a much more recent origin than Dicers, being present only in vertebrates, although homologues to their characteristic CAspase Recruitment Domains (CARDs) and RNA helicase domains are found in more basally branching deuterostomes, such as the tunicate *Ciona intestinalis* and the purple sea urchin *Strongylocentrotus purpuratus* [5, 7]. At present, direct viral sensing and immune induction functions have only been shown in vertebrates for two of the three RLRs, retinoic acid inducible gene I (RIG-I) [6] and melanoma differentiation associated gene 5 (MDA5) [8]. The third RLR, laboratory of genetics and physiology 2 (LGP2), binds viral RNA but cannot itself induce an immune response, instead triggering interferon production indirectly by signalling to MDA5 [9]. In contrast to the vertebrate-specific RLRs, the antiviral role of Dicer-like genes is much more widespread, being present in plants [10], fungi [11] and animals [12].

The Toll receptors were initially discovered in *Drosophila*, where they are involved in regulating the antibacterial and antifungal immune response [13]. The phylogenetic distribution (Figure 1) of Toll-like receptors (TLRs) suggests that they originated in the early Bilateria, before the divergence of protostomes and deuterostomes. In *Drosophila*, Toll-7 directly binds viruses and activates the autophagy response [14^••^]. In mammals, four TLRs (TLR3, 7, 8 and 9) play a pivotal role in sensing viral nucleic acids [15, 16, 17, 18], subsequently activating the innate and adaptive immune responses through IRF-3, IRF-7 and NF-κB [19]. Other mammalian TLRs recognise different PAMPs, including lipids (TLR1, 2, 4 and 6) [20, 21, 22] and proteins (TLR5) [23]. This phylogenetic distribution of antiviral function suggests that TLRs are likely to have evolved a viral sensing role early in animal evolution, before the divergence of the protostomes and deuterostomes.

## The evolution of RNA helicases

The most ancient conserved viral sensors are related to RNA helicases present in Archaea and Eukaryotes [5]. Two families of sensing helicases have been the subject of recent evolutionary study: the Dicers [24, 25••] and the Rig-I-like receptors (RLRs) [5, 7]. Two of the three RLRs (RIG-I and MDA5) each harbour two CARD domains that are integral in triggering the interferon response [6]. Despite this shared function, the two CARD domains appear to have substantially different histories [5], and it has therefore been suggested that the CARDs were gained by RIG-I and MDA5 in two separate events, with the first domain being acquired before the duplication that formed RIG-I and MDA5, and the second domain gained after they diverged [5]. Consistent with this, two CARD domains are found at separate loci in the sea anemone *Nematostella vectensis*, suggesting that the proposed grafting of these CARDs onto RLR may have occurred from these loci after the divergence of the chordates [7]. In contrast to the CARD domains, however, the order of divergence of RIG-I, MDA5 and LGP2 themselves remains unresolved. A neighbour-joining approach suggested that RIG-I diverged in the early deuterostomes, with LGP2 and MDA5 diverging later in the vertebrates [7], while Bayesian and Maximum Likelihood methods find that LGP2 diverged in the early chordates, with RIG-I and MDA5 diverging later in the tetrapods [5].

It is highly likely that the last eukaryotic common ancestor possessed one Dicer, which was duplicated to produce two paralogues in the early Metazoa soon after their divergence from the other eukaryotes [24, 25••]. However, the timing and extent of paralogue loss, and therefore the age of the two well-studied insect Dicer paralogues (Dicer1 & Dicer2), remains unresolved. It is possible that one of the paralogues was lost in the early Metazoa soon after the divergence of the Placazoa, and therefore Dicer1 and Dicer2 are relatively recent duplicates formed from a lineage-specific duplication in the ancestral arthropod [24]. Alternatively, large-scale lineage-specific loss of one of these paralogues may have left only the Placazoa and the arthropods with the two ancient paralogues [25^••^]. Reconstruction and rooting of this tree is made challenging by the extreme difference in evolutionary rate between Dicer1 and Dicer2, and by the high divergence to non-animal Dicers. Wider taxon sampling may mitigate these problems, and if so, then an ancient origin for Dicer1 and Dicer2 may be more likely [25^••^]. Accurate reconstruction of this phylogeny would help to determine the extent to which Dicer has retained its presumably ancestral antiviral role, which has been confirmed in plants, fungi, arthropods, and most recently mammals [26, 27].

Population-genetic approaches can be used to detect departures from a standard neutral model of evolution, and thus infer the action of recent or ongoing natural selection. These methods have been widely applied to Dicers and RLRs, and have utilised both within-species genetic diversity [28•, 29, 30, 31] and between-species divergence [1, 28•, 31, 32] to understand the role of positive selection in shaping these genes. In humans, RIG-I appears to be tightly constrained [31], possibly due to the broad range of viruses it detects [33]. In contrast, positive selection has been detected on human LGP2 and MDA5 [31], and may have driven selective sweeps of MDA5, with one variant fixing in Europe and Asia and an alternative variant selected in South America [30]. Across the mammals, positive selection has been detected at individual sites in all domains of RIG-I and MDA5, but only in the helicase domain of LGP2 [34]. Evidence for positive selection has also been found for *Drosophila* Dicer2, which evolves extremely rapidly [1] under strong positive selection [32]. Despite this, it remains challenging to confidently attribute these patterns of RLR evolution to virus-mediated natural selection, as there may be some other shared trait common to all members of the RLR gene family that may predispose them to evolve in this way. Nevertheless, as neither rapid evolution nor positive selection are detected for insect Dicer1 [32], a Dicer2-homologue in the microRNA pathway that lacks a major antiviral role, it seems likely that the rapid evolution of Dicer2 may be driven specifically by its viral sensing function.

## The evolution of the Toll-like receptors

All TLRs have characteristic leucine-rich repeat (LRR) and Toll/interleukin-1 receptor (TIR) domains, which function in PAMP recognition and cell signalling, respectively. These domains appear to have evolved separately in the early Metazoa, as a vertebrate-like TIR is present in the Cnidaria [35]. However, the combination of TIR and LRR domains is seen after the divergence of the Bilateria from basal Metazoa, but before the divergence of the protostomes and deuterostomes [35]. A similar age has been estimated for the TLR adaptor MyD88, which was identified in both vertebrates and invertebrates [36], and for the interaction between TLRs, MyD88 and NF-κB, which has been reported in the oyster *Crassostrea gigas* (Lophotrochozoa) [37^•^]. However, the full TLR signalling pathway appears to have been acquired slowly, as the other adaptors TIR domain-containing adaptor molecule (TICAM) and TIR domain-containing adaptor protein (TIRAP) appear first in the early chordates [38] following duplication of MyD88 [36].

Direct sensing of viral PAMPs also appears to have evolved in TLRs before the divergence of the protostomes and deuterostomes, being found in both *Drosophila* [14^••^] and vertebrates. Intriguingly, differential expression of TLRs occurs on exposure of *C. gigas* to different PAMPs [37^•^], suggesting that specialisation of TLR paralogues to specific classes of pathogens may also have occurred early in the Bilateria. Since its divergence from other deuterostomes, a dramatic expansion of the TLR gene family in the basal deuterostome *S. purpuratus* has produced 253 paralogues, some of which appear to have specialised to a larval-specific or antibacterial role [39]. However, whether any of these paralogues has an antiviral function, and therefore how viral sensing has influenced their evolution, remains unknown.

Studies of TLR molecular evolutionary dynamics have revealed that selective pressures vary between domains, between different levels in the TLR signalling pathway, and between TLRs with different functions. At the domain level, the LRR domain evolves much faster than the TIR domain [39, 40, 41, 42], consistent with the role of the latter in signalling to cytoplasmic adaptor molecules that are constrained by their interactions with multiple different TLRs. At the pathway level, a negative relationship between evolutionary rate and pathway position has been found in both *Drosophila* [43] and the Metazoa as a whole [44], suggesting that downstream components are under stronger purifying selection, possibly because of their interactions with multiple different upstream factors [44].

At the level of TLR function, four studies have explicitly compared the molecular evolutionary patterns of viral and non-viral TLRs in humans [45], rodents [46], primates [41], and mammals generally [47^••^]. These studies have used interspecific divergence at nonsynonymous and synonymous sites (d*N* and d*S*, respectively) to quantify the rate of protein evolution relative to the neutral expectation, with some studies going on to infer positive selection by testing for the existence of individual codon positions showing a d*N*/d*S* ratio greater than one. Comparisons that average d*N*/d*S* across the whole gene have all found that viral sensing TLRs evolve more slowly than TLRs that sense other pathogens; however, the magnitude of this difference in rates varies between focal lineages. In humans, viral sensing TLRs evolve much less rapidly than other TLRs, with average d*N*/d*S* values of 0.25 (viral) and 0.81 (non-viral) [45]. Far more modest differences have been found in rodents [46], primates [41], and birds [48]. Viral sensing TLRs may evolve more slowly because of stronger purifying selection, which has been detected using intraspecific polymorphism data from birds [48], humans [45] and primates as a whole [41]. Alternatively, the higher d*N*/d*S* ratio seen in TLRs that sense other PAMPs may reflect higher rates of positive selection, with a higher proportion of codons experiencing frequent adaptive substitutions.

**Figure 2:**
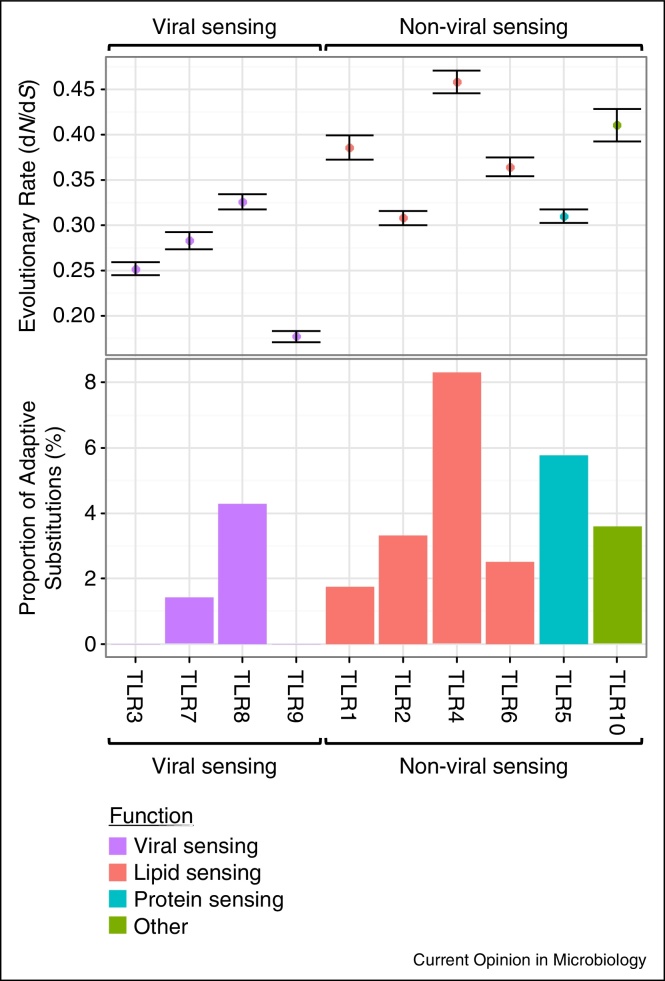
The evolutionary rate (d*N*/d*S* — upper panel) and the proportion of codons inferred to be positively selected (lower panel) in viral sensing and non-viral sensing TLRs across eight rodent and ten primate species. Sequences were obtained from GenBank, and their phylogeny reconstructed using the Bayesian phylogenetic analysis program MrBayes [49] (see Supplemental File 1 for alignment). Evolutionary rate was estimated under the M0 model in PAML [50] (error bars represent one S.E.), and the proportion of adaptive substitutions represents the estimated proportion of sites with d*N*/d*S* > 1 under the M8 model. Overall, it appears that the primate and rodent viral sensing TLRs evolve more slowly and have a lower proportion of adaptive substitutions than other TLRs.

Adaptive substitutions have been inferred both at the TIR and LRR domains and the TLR sequence as a whole. There is wide variation in the proportion of positively selected codons that are located in the PAMP-binding LRR region: this domain harboured all adaptive substitutions in rodents [46] and the majority in mammals [47^••^], but in primates this region contained none in viral sensing TLRs, and only a small minority in non-viral TLRs [41]. Across the whole sequence, a mammal-wide study failed to find a significant difference in the proportion of positively selected codons between viral and non-viral TLRs [47^••^]. However, individual studies of primates [41], rodents [46] and birds [48] identified fewer positively selected codons in viral sensing compared with non-viral TLRs. This may indicate that host-virus arms race dynamics exert a weak or negligible effect on viral sensing TLRs, perhaps because their membrane-bound location limits viral interference. Instead, their evolution may simply be constrained by the conserved nature of viral PAMPs, resulting in low rates of adaptation and few positively selected codons (illustrated in Figure 2).

## Conclusion

Viral sensors evolve under contrasting selective pressures: the conserved nature of viral PAMPs may tend to constrain evolution, whereas antagonistic host-virus coevolution may drive rapid evolution. The rapid evolution of RNA helicases could indicate that coevolution with other pathogen proteins (such as immune suppressors) is a major selective pressure on these sensors. In contrast, the slow evolution of TLRs may suggest the absence of a host-virus arms race acting directly on the sensor. In the future, this could be tested by further investigation of viral immune suppression strategies, and the overall importance of such strategies in shaping evolution could be informed by comparative studies of the evolution of viral sensors in a broader phylogenetic range of taxa.

## References and recommended reading

Papers of particular interest, published within the period of review, have been highlighted as:

• of special interest
•• of outstanding interest
